# Emotional Risk Factors, Rumination, and Self-Criticism in Relation to Suicidal Ideation Among Chinese Depressive Outpatients

**DOI:** 10.3390/bs14111111

**Published:** 2024-11-19

**Authors:** Barbara Chuen Yee Lo, Sophie Kai Lam Cheng

**Affiliations:** 1 School of Educational Psychology and Counselling, Monash University, Melbourne 3800, Australia; 2 Department of Psychology, The Chinese University of Hong Kong, Hong Kong SAR, China

**Keywords:** suicidal ideation, depression, rumination, self-criticism

## Abstract

Previous studies indicated that individuals with major depressive disorder (MDD) are at high risk of suicide, making the identification of risk factors in suicidal depressive outpatients essential for developing effective prevention and treatment strategies. This study aims to (1) identify emotional risk factors and (2) examine the relationship between cognitive markers—including rumination and self-criticism—and suicidal ideation among depressive outpatients. A total of 165 Chinese clinical outpatients with depression were recruited from local hospitals in Hong Kong. In our sample, 68% of patients with moderate depression and 87.5% of patients with severe depression reported experiencing suicidal thoughts. The results from the logistic regression revealed a significant interaction between rumination and self-critical thoughts in relation to suicidal ideation. Specifically, the ruminative patients with self-critical thoughts were more likely to have suicidal thoughts than those without self-critical thoughts. These findings align with the cognitive model of suicide, suggesting that maladaptive information processing and negative cognitive content are associated with an increased risk of suicide. The current findings have clinical implications for the future development of more specific and accurate mental health assessment, preventive programs, and psychotherapies for depressive outpatients.

## 1. Introduction

### 1.1. Depression and Suicidal Ideation

Suicide is a leading cause of death worldwide, with more than 700,000 people dying by suicide every year [1]. Among various risk factors, suicidal ideation is one of the strongest predictors of subsequent suicidal behavior [2]. Individuals’ suicidal ideation includes thoughts, images, beliefs, voices, or other cognitions about intentionally ending their life [3].

Previous studies suggested that major depressive disorder (MDD) and depressive symptoms are major risk factors for suicidal ideation and are significantly associated with increased suicidal thoughts [4]. People with depression experience depressed mood, loss of interest or pleasure in activities, fatigue or energy loss, etc. [5]. Over a quarter of those who die by suicide with major depression are in contact with psychiatric services at the time of their death in the UK [6], indicating that even though the patients were receiving regular mental health services or had help-seeking behaviors, they were still potentially in danger of committing suicide. Therefore, the identification of key risk factors for suicide in individuals with depression is essential if clinicians are to identify those most at risk and intervene appropriately. There is good evidence that monitoring and active treatment for high-risk patients may result in reduced suicide rates [7]. Furthermore, there is a rapid transition from suicidal ideation to plans and attempts. As Borges et al. [8] suggested, intervention efforts need “to focus on prevention of ideation rather than prevention of the transition from ideation to more serious outcomes” (p. 73). Therefore, prevention of ideation could be more critical and effective in reducing suicides than treatment after suicidal thoughts have occurred.

### 1.2. Other Risk Factors and Intervention of Suicidal Ideation

Research has identified a number of factors implicated in the etiology and course of suicidal ideation. Sociodemographic characteristics include being male, being unmarried, low income, unemployment, low levels of education, and living alone [4,9,10]. In addition to demographic factors, assessments of emotional and cognitive factors have been conducted. The emotional factors include depression, anxiety, stress, worry, etc. [11]. For instance, studies found that patients with anxiety, which is characterized by feelings of tension, worried thoughts, and physical changes like increased blood pressure [12], were more likely to have suicidal ideation compared to those without anxiety [13]. In addition, the cognitive factors include cognitive rigidity, rumination, worry, attentional biases, etc. [14,15].

Among the risk factors, maladaptive cognitive perception has been studied as a transdiagnostic marker of suicide risk. Lazarus’s Psychological Stress Theory [16] suggests that people’s cognitive perception and appraisal of stress is related to the way they cope with difficulties. It is not the situation, but the way in which a person interprets the situation, that affects the person’s experience [17]. Studies found that perceived stress, shaped by how individuals appraise situations, strongly correlates with mental health outcomes, including depression [18] and suicidal ideation [19]. A study conducted among sexual minorities also found that perceived stress is significantly associated with suicidal ideation, suicide attempt, and self-harm [20]. When people experience harm, loss, threat or challenge, the way in which they appraise the situation can affect the way they react to it, indicating the importance of studying cognitive factors in understanding the development of suicide risk.

Furthermore, many evidence-based therapies for preventing suicide include cognitive components, such as cognitive behavioral therapy (CBT), mindfulness-based cognitive therapy (MBCT), dialectical behavior therapy (DBT), problem-solving therapy, mentalization-based treatment, and psychodynamic interpersonal therapy [21]. A recently developed Psychospiritual Meaning Intervention (PSMI) [22], targeting suicide ideation and depression, also tended to focus on thoughts and the ways in which people handle emotions and stress. These interventions were found to be effective in reducing suicidal thoughts and behaviors. Since cognition is central to these interventions, understanding specific cognitive factors could provide more details for and enhance the specificity of treatment plans. It would also offer practical implications for clinical psychologists and mental health professionals to apply more effective and tailored therapeutic techniques for patients.

### 1.3. Rumination and Suicidal Ideation

Several models of suicide highlight the role of negative cognitive content (e.g., cognitive distortions, aversive cognitions) and processes (e.g., maladaptive rumination) associated with psychiatric disturbance in activating suicidal crisis [23,24]. For example, Rudd’s [25] cognitive-behavioral model of suicidality assumed that the central pathway for suicidality is cognition. That is, maladaptive negative perceptions and beliefs play a central role in influencing suicide risk along a continuum from ideation to action. Individuals with cognitive vulnerability can be predisposed to suicidality. Furthermore, according to the cognitive model of suicide, when individuals undergo continued stress, their maladaptive cognitive process is associated with a higher likelihood of being in a suicidal crisis [24]. The maladaptive cognitive process includes two components, namely maladaptive information processing and negative cognitive content. One specific information processing style that has received much attention regarding its correlation to concurrent and prospective suicidal ideation is rumination (see [26] for a meta-analytic review). Rumination as a maladaptive style of processing emotion, involves repetitive thinking about the causes, situational factors, and consequences of negative emotional experiences [27]. It is also a significant factor that contributes to the maintenance of depression [28]. A recent one-year longitudinal study found that rumination is associated with suicidal ideation and attempts among individuals with MDD [29].

In addition to information processing, different cognitive contents could be associated with different behavioral outcomes. Previous studies have found some mixed findings on the relationship between rumination and suicide. While studies showed that all forms of rumination were related to severity of suicidal ideation [30], some studies found that when rumination has different thinking content, such as focusing on episodes of anger or revenge fantasies, it has no significant relation with suicidal ideation or attempts [31]. This inconsistency implies the possibilities of cognitive content as potential moderators in the association between rumination and suicidal ideation.

### 1.4. Self-Criticism as a Moderator

While negative cognitive contents and biased information processing have been widely studied in the context of suicidality and depression, these two components are often studied separately. Hence, one may wonder how they interact with one another to affect suicide ideation. As individuals who are ruminating remain fixated on stressful events and on their negative emotions about these problems [32], it is of great significance to explore the negative cognitive contents they might have that may contribute to serious suicidal outcomes.

Self-criticism, which is characterized by setting high and possibly unrealistic self-standards and adopting a judgmental and punitive stance toward oneself [33], was found to be associated with mental health disorders, including depression, and can directly linked to suicide [34,35]. Previous studies found that self-criticism is associated with a relative intolerance to one’s failure to attain the standard one has set [36]. According to the strain theory of suicide, when individuals are experiencing psychological strain, which is defined as the mental torment resulting from conflicting and competing pressures, are more likely to conduct suicide [37]. Specifically, when they struggle with aspiration strain, individuals have a discrepancy between their aspiration or high goal and their reality, which in turn leads to frustration and pain. Studies of suicide across different countries showed that psychological strains were significantly associated with suicide to different extents [38,39]. In addition, O’Neill et al. [35] claim that self-criticism may fulfil the role of an ongoing stressor that contributes to negative perceptions of self, which can in turn lead to self-attack, self-harm, depression, and even suicide [40,41].

Although previous studies revealed that both self-criticism [35] and rumination [26] play important roles in the development of suicide risk, their interactive effect on suicidal ideation remains unclear. As mentioned previously, not all ruminative individuals may develop suicidal thoughts, but the content that they are thinking can intensify the related emotion in the process of rumination [42]. Specifically, self-critical individuals are intolerant of their failure, feel shame about themselves, and engage in judgmental thinking when they experience a discrepancy between their goals and reality. Consequently, when the rumination process is dominated by self-critical thoughts, people tend to repeatedly focus on negative self-judgements and become trapped in aspiration strain, which increases the likelihood of developing suicidal ideation. Indeed, previous studies found that when attention is focused specifically on self-critical thoughts, rumination is significantly associated with depression and acute distress [42,43].

By examining the moderating role of self-criticism in the link between rumination and suicidal ideation, this study could contribute to the theoretical understanding of cognitive processes and refine cognitive theories related to suicidality. It may also inspire further research into how cognitive interactions affect mental health outcomes, promoting a more nuanced understanding of the complex dynamics at play in suicidal ideation.

### 1.5. Current Study

The present study aims to (1) understand the emotional and cognitive factors that are associated with suicidal ideation among Chinese depressive outpatients, including depression, anxiety and stress, worry, rumination, and self-criticism; and (2) study the interactive effect between two cognitive factors, rumination (a maladaptive form of information processing) and self-criticism (a form of negative cognitive content). In the path from maladaptive information processing to suicidal ideation, the relationship between rumination and suicidal thoughts can be moderated by cognitive factors such as self-criticism. We hypothesized an interaction between rumination and self-criticism in affecting suicidal ideation among depressive outpatients. Specifically, ruminative depressive patients who have self-critical thoughts would be more likely to have suicidal ideation than those who do not have self-critical thoughts.

Given the high prevalence of suicide mortality each year, it is critical to identify and understand the risk factors that are linked to suicidal ideation in order to provide effective suicidal prevention treatment and reduce suicidal behaviors in society. These insights can aid in designing preventive community-based programs that address high-risk cognitive patterns, potentially reducing the incidence of suicide in society and improving mental health outcomes overall.

## 2. Materials and Methods

### 2.1. Study Type and Design

The current study is a cross-sectional study to understand the profile of various psychological and cognitive variables in a clinical sample with suicidal ideation and examine the relationship between cognitive factors and suicidal ideation. Data were collected at a single point from a group of depressive outpatients to reduce burden for participants. By focusing on associations between the variables, this study yields important insights into which psychological characteristics and cognitive patterns are most closely related to suicidal ideation.

### 2.2. Participants and Procedure

One hundred and sixty-five clinical outpatients (136 females and 29 males) aged from 19 to 65 years (M = 45.21, SD = 10.46) were recruited in Hong Kong by convenient sampling. All of them were ethnic Chinese and were outpatients who were experiencing or had previously had major depressive episodes (MDE). Before the study, they were assured of their right to refuse to participate and to withdraw from the study at any time without any consequences. They were also informed that a trained, experienced researcher would contact them to conduct a structured interview and that all data collected would be kept confidential. The research assistants had been well trained by a clinical psychologist for over 15 h to conduct the interviews by following the Structured Clinical Interview for DSM Disorders (SCID), which is a semi-structured interview guide for making major DSM diagnoses. The interview was conducted by the research assistant individually with each participant in a private room at the hospital. To ensure the uniformity of the interview procedure, a clinical psychologist randomly sat in several interview sessions to evaluate the research assistant’s performance, confirmed that the procedure followed established standards, and provided supervision afterwards.

### 2.3. Measures

#### 2.3.1. Suicidal Ideation and Self-Criticism

Suicidal ideation and self-critical thoughts were assessed by using the questions derived from the Beck Depression Inventory—Second Edition (BDI-II) [44]. Participants were asked to choose one statement that best described the way they had been feeling during the past two weeks from groups of suicidal thoughts and self-criticism. Participants who chose “I don’t have any thoughts of killing myself” were grouped as non-suicidal group, and those who chose a non-zero score, including “I have thoughts of killing myself, but I would not carry them out”, “I would like to kill myself”, or “I would kill myself if I had the chance” were coded as having suicidal intention. The suicide item of the BDI has been validated as an appropriate screening tool for both future suicidal attempts and suicides in prospective cohorts [45]. Self-critical thoughts were coded in the same way, in that participants who selected “I don’t criticize or blame myself more than usual” were coded as not having self-critical thoughts. The Chinese version of the BDI has good reliability, since the Cronbach’s α was 0.94 for clinical samples and 0.88–0.94 for non-clinical samples [46].

#### 2.3.2. Depression

The 20-item Center for Epidemiological Studies Depression scale (CES-D) [47] was implemented and participants were asked to rate how often over the past week they had experienced symptoms associated with depression. Response options ranged from 0 to 3 for each item (0 = rarely or none of the time, 1 = some or a little of the time, 2 = occasionally or a moderate amount of the time, and 3 = most or all of the time). Scores ranged from 0 to 60, with high scores indicating greater depressive symptoms. Patients were categorized into one of the following three groups: (a) no or minimal depression (0–19 points); (b) moderate depression (20–39); (c) severe depression (more than 40 points). A cut-off score of 20 was used to classify patients with depressive symptoms, with good sensitivity and specificity [48]. The Chinese version of CES-D has been tested among Chinese populations and has showed adequate construct validity and good reliability (Cronbach α = 0.82–0.95) [49,50]. In the present sample, Cronbach α was 0.91, which shows high internal consistency.

#### 2.3.3. Anxiety and Stress

Anxiety and stress were assessed by using the anxiety subscale and stress subscale, respectively, from the 21-item Depression Anxiety Stress Scales (DASS-21) [51]. Each subscale consists of 7 items. Participants were asked to use 4-point severity/frequency scales ranging from 0 (not at all) to 3 (most of the time) to rate the extent to which they had experienced each state over the past week. The DASS-21 has been translated into different languages, including traditional Chinese, and is widely accessible to researchers around the globe. Previous studies have supported the psychometric characteristics and utility of Chinese version, including good structural validity and reliability [52,53]. In the current sample, the DASS-21 also shows high internal consistency, with Cronbach α equal to 0.95.

#### 2.3.4. Worry

The trait of worry was measured by the Penn State Worry Questionnaire (PSWQ) [54], which is a 16-item self-report scale. Participants were asked to choose a number that best describes how typical or characteristic each item is for them on a 5-point Likert scale (1= not at all typical, 5 = very typical of me). A sample item is “My worries overwhelm me”. Scores range from 16 to 80, with higher scores indicative of higher levels of trait worry. The Chinese version of PSWQ has been validated and possesses adequate convergent and discriminate validity and good reliability [55]. Our results also showed good internal consistency (Cronbach’s α = 0.88).

#### 2.3.5. Rumination

The 10-item Ruminative Response Scale (RRS) [56] was used to measure the severity of depressive rumination. Each item indicates the frequency of each event by rating a four-point Likert scale ranging from 1 (almost never) to 4 (almost always). The scale has 2 factors, brooding and reflection, with 5 items measuring brooding, and 5 items measuring reflection. The total score ranges from 10 to 40, with higher scores indicating a greater tendency to ruminate. The scale has demonstrated sound test–retest reliability, discriminant validity, and convergent validity [56]. The Chinese version of the scale was also reliable with good internal consistency (Cronbach’s α = 0.82) and valid in examining rumination [57]. Cronbach’s α coefficients in our study were 0.84 for total scale.

### 2.4. Statistical Analyses

All statistical analyses were performed using IBM SPSS software version 27.0 [58]. The descriptive statistics of variables were analyzed to understand the difference between the suicidal and non-suicidal groups. A paired independent sample *t*-test was conducted to compare the characteristics of the outpatients who had or did not have suicidal thoughts. The interaction and moderating effect between cognitive factors were further tested in binary logistic regression models. The level of significance was set at 5% in all statistical tests, and a 95% confidence interval (CI) accompanied each estimate, where appropriate.

## 3. Results

### 3.1. Emotional and Cognitive Factors Associated with Suicidal Ideation

Table 1 presents the participants’ characteristics and the *t*-test or chi-squared test results on the differences between suicidal and non-suicidal group. Of the participants, 94 (57%) of them reported having suicidal thoughts, among whom 84% were female. The suicidal group showed significantly greater depression (CES-D) (*t* = 8.34, *p* < 0.001), more severe anxiety (DASS—Anxiety) (*t* = 5.89, *p* < 0.001), more stress (DASS—Stress) (*t* = 6.92, *p* < 0.001), more worries (PSWQ) (*t* = 2.73, *p* < 0.001), and higher levels of rumination (RRS) (*t* = 4.93, *p* < 0.001) than the non-suicidal group. Regarding the perceived depression levels of the participants, more than a quarter of the participants (30.3%) reported minimal depression, and more than half of the participants (55.2%) reported moderate levels of depression. Some of them (14.5%) perceived themselves as suffering from severe depression. A significantly higher proportion of those who had suicidal ideation experienced severe depression relative to those who had not (22.3% vs. 4.2%; *χ*^2^ = 42.58, *p* < 0.001).

### 3.2. The Interactive Effect Between Rumination and Self-Criticism

Table 2 reports the results of the structured logistic regression model indicating the factors relating to suicidal ideation in clinical depressive outpatients. The logistic regression model was statistically significant, with *χ*^2^ = 53.67, *p* < 0.001. The model explained 37.3% (Nagelkerke *R*^2^) of the variance in suicidal intention and correctly classified 77.0% of cases. After controlling for other variables, the logistic regression model results indicated that the rumination × self-critical thoughts interaction effect was significant (*p* < 0.01), indicating the moderating role of self-critical thoughts in the relationship between rumination and suicidal ideation. The results showed that ruminative patients with self-critical thoughts were associated with an increased likelihood of having suicidal thoughts (adjusted odds ratio (aOR) = 1.13, *p* < 0.01) compared to those without self-critical thoughts.

## 4. Discussion

The present study explored the differences between depressed outpatients with and without suicidal thoughts, and examined emotional and cognitive risk factors related to their suicidal ideation. In particular, our *t*-test findings indicated significantly higher levels of depression, anxiety, stress, worries, and rumination in the suicidal group compared to the non-suicidal group. These are consistent with previous research observing that symptoms of depression, anxiety, worry, and maladaptive emotional self-regulatory responses, such as rumination, were significantly associated with suicide ideation or attempts [15,59]. In addition, we focused on the cognitive process underlying the suicidal ideation among depressive outpatients. By drawing together the strain theory of suicide [37] and the cognitive model of suicide [24], this study proposed and tested the moderating role of self-criticism in the relationship between rumination and suicidal ideation. The results supported our hypothesis that self-criticism moderates the relationship between rumination and suicidal ideation among depressive outpatients. Specifically, ruminative outpatients with self-critical thoughts have a higher risk of having suicidal ideation than those without self-critical thoughts.

Our study supports the research direction of investigating the interplay between cognitive processing and maladaptive thoughts. Wenzel and Beck [24] have proposed a cognitive model of suicide suggesting that maladaptive cognitive processes, including both maladaptive information processing and negative cognitive content, are associated with higher risk of being in a suicidal crisis. The model pointed out that cognitive processes, which include maladaptive information processing (i.e., how people are thinking) and cognitive content (i.e., what people are thinking), could lead to overwhelming distress and culminate in suicidal acts. Over the past few decades, research concerning the role of rumination in psychopathology has grown considerably. Rumination as a maladaptive form of information processing has been implicated in mood disorders, particularly depression [60] and in the prediction of suicidal ideation and attempts among Chinese individuals with depression [29]. A recent systematic review also indicates that rumination was significantly correlated with increased suicidal ideation in depressive disorders (r = 0.30, 95% CI (0.21, 0.38), *p* < 0.01) [61]. Nevertheless, previous research was largely focused on how rumination leads to suicidality, and the question of which thoughts can influence this relationship remains unclear. The current study extended past studies to examine the role of self-criticism in influencing suicidal thoughts among high ruminators.

Our finding echoes the finding in the past literature that self-criticism acts as a moderator in increasing the risk of suicidal ideation. A study conducted among community samples in Portugal found that distressed individuals have greater severity of suicidal ideation when they have high levels of self-criticism compared to those with low levels of self-criticism [62]. It highlighted the important role of self-criticism in suicidal ideation when people have high levels of distress. The current study found that self-criticism and rumination can interact to increase the likelihood of having suicidal ideation among depressive patients. People who have self-critical thoughts tend to aim for perfection in the self, set unreasonably high standards and heighten concern over errors, which are associated with depressive symptoms [63]. According to the strain theory of suicide, people who have high expectations and goals but fail to reach them in reality develop aspiration strain, which forms one of the risks leading to suicide [37]. Therefore, if high ruminators are preoccupied by their self-critical thoughts, they are more likely to repetitively think about the negative judgments they form towards themselves and be overwhelmingly dissatisfied with themselves, which in turn increases the risk of suicidal ideation.

### 4.1. Clinical Implications

While major depressive disorder (MDD) has been identified as a risk factor for suicide, it is clear that not every depressed patient is subject to having suicidal intent. Our study highlights the importance of (i) identifying relevant cognitive vulnerability markers and (ii) understanding the complexity of a dynamic, interactive process that underlies depressive suicidality. Specifically, high rumination and self-criticism were found to be associated with suicide risk among depressive outpatients in the present study, and this helps to inform health care professionals to remain alert to these individual characteristics in mental health assessment in applied contexts. This awareness allows for earlier intervention and, potentially, more accurate monitoring, which could prevent escalation. Understanding the cognitive risk factors also guides the development of preventive and psychoeducation programs, potentially reducing the incidence of suicidality in this group. For instance, preventive strategies, such as including skills training to address rumination, perfectionism, and judgmental self-evaluations. can be emphasized. Furthermore, personalized treatments, such as cognitive-behavioral therapy (CBT) and mindfulness-based cognitive therapy (MBCT) which focused on self-compassion and self-acceptance, may also address self-critical thoughts in practice to enhance treatment effectiveness.

### 4.2. Limitations and Directions for Future Studies

Although the current study has some important implications and strengths, some limitations are worth noting. Firstly, since our research was cross-sectionally designed, no causal linkage can be established among our studied variables. Secondly, it is worth mentioning that the measurement of the variables was based on self-reporting, which can be biased due to social desirability. In addition, we used a single item to screen for suicidal ideation, since single-item surveys were widely used in previous studies to assess various constructs, and they are efficient and less burdensome for patients to complete in clinical studies. However, future studies may consider adding more comprehensive assessments in evaluating this research variable. Thirdly, the sample in the study had an unbalanced male–female ratio, which is predominantly female (82.4%). One should therefore be cautious when interpreting or comparing these results. Fourthly, this study only focused on self-criticism and the aspiration strain proposed by the strain theory of suicide. Past research has indicated that some cognitive factors, such as feelings of shame and guilt, are significant risk factors for suicidality among mental health outpatients [64]. It is also found that hope can be a moderator to weaken the relationship between rumination and suicidal ideation [65]. To fully understand the relationship between these cognitive factors and suicidal ideation, more research is warranted to explore other possible moderators in the relationship between rumination and suicidality.

The present study examined the interaction effect of cognitive factors among Chinese participants. It is noteworthy that cultural background may also be a potential factor that affects the level of self-criticism and its association with suicidal ideation. Asian cultures, in general, represent a heterogeneous mixture of values. In this study, our sample was composed of ethnic Chinese individuals. Chinese culture is broadly categorized as collectivistic, with an interdependent form of self-construal that emphasizes group harmony and fitting in with social norms [66]. Individuals adopting these values tend to engage in social comparison to identify personal shortcomings and pursue self-improvement [67]. Studies conducted in other interdependent cultures, such as Japan, indicate that self-criticism is prevalent within collectivistic frameworks [68]. Parents in China [69] and South Korea [70] who adopt interdependent self-construal also tend to be more critical of their children and have high expectations towards their children. The inability to meet the expectations of their parents can give self-critical individuals additional stress, making them feel that they are a burden, which in turn leads to suicide ideation [70]. However, a study conducted among Filipino individuals showed different findings, suggesting that the relationship between self-criticism and depressive symptoms was weaker [67]. Self-criticism may have an adaptive function to help individuals to accurately evaluate whether their behavior is in accordance with unwritten social rules and expectations [71]. Future studies may further explore cultural differences by conducting cross-cultural research to understand the moderating role of self-criticism in the relationship between rumination and suicidal ideation.

## 5. Conclusions

To conclude, this study sheds light on the association between rumination and suicidal ideation among Chinese depressive outpatients. The findings suggest that self-criticism plays a moderating role in this relationship, highlighting the importance of considering maladaptive cognitive factors in the development of suicidal ideation. Specifically, ruminative depressive outpatients who tend to self-criticize have a higher likelihood of developing suicidal thoughts. These results have important implications for the assessment and treatment of suicidal ideation in depressive outpatients. Further research is needed to replicate and extend these findings, with a focus on identifying effective interventions that can target these processes and reduce the risk of suicidal ideation among individuals with depression.

## Figures and Tables

**Table 1:** Descriptive Statistics of Outpatients With/Without Suicide Risk and Cronbach’s α of Scales.

			With/Without Suicide Risk				
	Overall (%)		With Risk (%)		Without Risk (%)		
	M	SD	M	SD	M	SD	*t*/*χ*^2^
Gender							0.40
-Female (N, %)	136 (82.4)		79 (84.0)		57 (80.3)		
-Male (N, %)	29 (17.6)		15 (16.0)		14 (19.7)		
CES-D (α = 0.91)	27.68	11.17	32.97	9.61	20.68	9.06	8.34 **
-Minimal (N)	44 (26.7)		7 (7.4)		37 (52.1)		
-Moderate (N)	97 (58.8)		66 (70.2)		31 (43.7)		
-Severe (N)	24 (14.5)		21 (22.3)		3 (4.2)		
DASS_Total (α = 0.95)	51.23	27.63	63.33	24.02	35.14	23.72	7.46 **
-DASS_Depression	16.82	10.84	21.48	10.01	10.63	8.60	7.27 **
-DASS_Anxiety	14.97	9.27	18.34	8.51	10.49	8.33	5.89 **
-DASS_Stress	19.44	9.83	23.51	8.65	14.03	8.67	6.92 **
PSWQ (α = 0.88)	54.72	12.47	57.00	12.85	51.71	11.36	2.73 *
RRS (α = 0.84)	21.63	5.61	23.38	5.00	19.31	5.57	4.93 **

Note. CES-D = Center for Epidemiologic Studies Depression; DASS = Depression Anxiety Stress Scales; PSWQ = Penn State Worry Questionnaire; RRS = Ruminative Response Scale. * *p* < 0.01, ** *p* < 0.001.

**Table 2:** A Logistic Regression Model Predicting Suicide Risk in Clinical Outpatients.

Variables	Coef (B)	S.E.	b	aOR	95% CI	Sig.
Gender						
-Female	−0.18	0.50	−0.02	0.84	0.31–2.25	0.73
-Male	--	--		1.00	--	--
Self-criticism						
-With self-critical thoughts	−2.93	1.66	−0.32	0.05	0.00–1.38	0.08
-Without self-critical thoughts	--	--		1.00	--	--
RRS	0.01	0.05	0.02	1.01	0.92–1.12	0.78
RRS X Self-criticism	0.23	0.08		1.26 *	1.07–1.47	0.00
Goodness of fit test	Test statistics				Sig.	
*χ* ^2^	53.67 **				<0.001	
Hosmer–Lameshow	7.36				0.50	
Cox and Snell *R*^2^	0.278				--	
Nagelkerke *R*^2^	0.373				--	

Note. RRS = Ruminative Response Scale; aOR = adjusted odds ratio; CI = confidence interval. * *p* < 0.01, ** *p* < 0.001.

## Data Availability

The data that support the findings of this study are available from the corresponding author upon reasonable request.
